# Potential Risk for Hearing from Prolonged Exposure to Sound at Conversation Levels

**DOI:** 10.3390/audiolres16030076

**Published:** 2026-05-22

**Authors:** Wenyue Xue, Nolan Sun, Emily Wood, Jason Xie, Xiuping Liu, Jun Yan

**Affiliations:** Department of Physiology and Pharmacology, Hotchkiss Brain Institute, Cumming School of Medicine, University of Calgary, Calgary, AB T2N 4N1, Canada

**Keywords:** low-level noise, hidden hearing loss, ABR, hearing health

## Abstract

**Background:** Prolonged exposure to moderate and loud noise is known to impair hearing; however, the safety of long-duration exposure to low-level sound, such as that encountered during everyday conversation, remains unclear. This study aimed to determine the effect of continuous exposure to sound at a 65 dB sound pressure level (SPL) on auditory processing. **Methods:** Auditory brainstem responses (ABRs) were recorded in C57BL/6 mice before and after a 1 h exposure to a continuous pure tone at 65 dB SPL. Changes in ABR thresholds, wave amplitudes, and latencies were analyzed across frequencies and time points. Correlations between amplitude and latency changes across ABR waves were also assessed. **Results:** Tone exposure induced a significant, frequency-specific increase in ABR thresholds, with a mean elevation of approximately 6 dB and a maximum shift of 15 dB. Significant reductions in amplitudes and prolongations of latencies were observed in Waves I–III, while Wave V amplitude remained relatively stable. A strong negative correlation between amplitude reduction and latency increase was found in Wave I, which progressively weakened from Wave II to Wave V. These functional changes persisted for up to three hours following exposure before gradually returning to baseline. **Conclusions:** Prolonged exposure to low-level sound at intensities typical of conversational speech can transiently impair auditory function and alter early neural processing in the auditory pathway. These findings suggest that sound levels commonly considered safe may still pose a risk when exposure is sustained, with implications for understanding hidden hearing loss and improving early diagnostic approaches.

## 1. Introduction

Sound, as a medium of daily communication, can be hazardous to hearing depending on its intensity and duration of exposure. A specific type of acquired sensorineural hearing loss, known as hidden hearing loss (HHL), has attracted increasing attention [1,2,3]. HHL refers to hearing difficulty experienced by individuals who show normal hearing sensitivity on an audiogram and have no history of exposure to loud sound (>105 dB SPL) or ototoxic drug use [4,5]. Prolonged exposure to low-level sound is believed to be one of the causes of HHL [6,7].

Exposure to loud sound for a sufficient duration can cause temporary or permanent hearing loss (i.e., decreased sensitivity or increased hearing thresholds). Sounds at or below 80 dBA for 8 h per day (Leq,8 h = 80 dBA) are generally considered safe. However, prolonged exposure to moderate sound (Leq,8 h= 80 dB SPL to Leq,2 h = 105 dB SPL) is postulated to cause HHL and has been studied in animal models for some time [6,8,9,10]. Multiple studies have shown that prolonged exposure to moderate sound leads to significant changes in the peripheral and central auditory system, with recoverable hearing thresholds [11,12,13]. For example, exposing mice to Leq,2 h = 95–98 dB SPL results in progressive synaptopathy, characterized by the loss of presynaptic ribbons and swelling of the auditory nerve terminals in the inner ear [14]. Functionally, exposing rats to Leq,2 h = 97 dB SPL increases hearing thresholds by up to 16 dB [15]. Significant alterations in neural information processing have also been observed in the auditory cortex and midbrain [16,17].

The question raised here centers on the impact of exposure to repetitive or prolonged sound at the acoustic level in the living environment, such as noise inside a moving car (typically 70–90 dBA) or from personal listening devices (recommended level of <70 dBA) [18,19]. To date, only scattered studies have investigated the effects of sound levels below 80 dB SPL. Zhou and Merzenich reported that exposing mice to noise bursts (Leq,24 h = 65 dB SPL) for 2 months led to decreased performance in temporal rate discrimination tasks without threshold increase in ABR [20]. Lau and colleagues, using functional magnetic resonance imaging, demonstrated a level-dependent decrease in blood oxygenation in the rat medial geniculate body and auditory cortex following a 2-month broadband noise exposure (Leq,24 h = 65 dB SPL). In contrast, Liu and colleagues recently reported a significant elevation in the threshold of cochlear compound action potential of rats following exposure to a banded continuous noise (18–24 kHz) at 65 dB SPL for 6 weeks, without a significant change in the function of the inferior colliculus [21]. These studies prompted our investigation into whether exposure to ‘safe’ sound can potentially induce HHL.

To clarify the impact of ‘safe’ sound on hearing, we examined the changes in mouse ABR following a 1 h exposure to a persistent tone at a level of 65 dB SPL (TE_65_). Our work showed a significant increase in ABR thresholds, a decrease in the amplitude of Wave I (as represented in the activity of the auditory nerve and cochlear nucleus), and, finally, the down-regulated correlation of changes in latency and amplitude from Wave I through to Wave V.

## 2. Methods

### 2.1. Animals and Anesthetics

Mice (C57BL/6, 5–7 weeks old, 16.8–21.5g) used in this study were obtained from the Charles River Laboratories (Senneville, QC, Canada). They were housed at the Animal Resource Centre, University of Calgary (UofC), in cages where they were exposed to a 12 h light/dark cycle, and given free access to water and food. The experimental protocol followed was approved by the Animal Care Committee (UofC, protocol no.: AC22-0115, 12 September 2022). Mice with a hearing threshold higher than 30 dB SPL were excluded.

For the 8 h experimental procedure, only female mice (*n* = 30) were selected to maximize physiological stability. This reduces the risk of anesthesia-induced hypoxia, which can confound hearing threshold measurements or increase mortality. Mice were anesthetized with an intraperitoneal injection of 85 mg/kg ketamine (Vétoquinol N.-A. Inc., Lavaltrie, QC, Canada) and 15 mg/kg xylazine (Vétoquinol N.-A. Inc., Lavaltrie, QC, Canada). The anesthetic level was maintained throughout the physiological experiment, including the tone exposure period. The anesthetic depth was monitored every 20 min by paw pinching. Additional dosages of ketamine (17 mg/kg) and xylazine (3 mg/kg) were administered when the mouse showed any withdrawal responses. The mouse’s head was fixed using a head holder by clamping the palate and nasal/frontal bones, with alignment of the bregma and lambda in the same horizontal plane. Body temperature was maintained at ~37 °C through a feedback-controlled heating pad. All experiments were conducted in an echo-attenuation soundproof chamber.

### 2.2. Acoustic Stimulation and Tone Exposure (TE_65_)

Tone bursts (10 ms long with 1 ms rise/fall time) were used as acoustic stimuli for sampling ABR. A 1 h continuous tone at 65 dB SPL (TE_65_) was used for sound exposure. These acoustic signals were digitally generated and converted into analog signals using an Enhanced Real-time Processor (RP2, Tucker–Davis Tech., Gainesville, FL, USA). Tone was delivered via a digital attenuator (PA5, Tucker–Davis Tech., Gainesville, FL, USA) to a loudspeaker placed 20 cm away, 45 degrees lateral to the mouse’s right ear. The output of the loudspeaker, calibrated for the location corresponding to the right ear of the mouse, is expressed as the decibel sound pressure level (dB SPL) within 1 decibel of accuracy (reference 20 μPa).

### 2.3. Tone Exposure (TE_65_)

Under anesthesia, mice were exposed to a continuous pure tone for 1 h. The tone was delivered to the right ear of the mouse through the loudspeaker. The tone frequency was set to the best frequency that was determined by the ABR receptive field (see below) and the tone level was set at 65 dB SPL (a typical conversation level). To ensure unilateral exposure, the contralateral ear canal was blocked with warm saline drops prior to tone delivery and cleaned afterward.

### 2.4. ABR Recording

To record the ABR, two stainless steel electrodes were placed subcutaneously, one at the vertex (~1 mm posterior to the lambda point) and the other at the mastoid (below the pinna of the right ear). Bioelectrical signals were amplified 5000 times, filtered with a bandpass of 0.25–2.5 kHz, and then fed to an Enhanced Real-time Processor (RP2, Tucker–Davis Tech., Gainesville, FL, USA) for data acquisition (BrainWare 9.22, Tucker–Davis Tech., Gainesville, FL, USA). Tone-evoked ABR potentials were sampled in a time window of 20 ms, starting from the tone onset, and ABR data were obtained by averaging 500 traces in response to an identical tone.

ABR receptive fields were obtained by recording ABR in response to a set of tones with various frequencies and levels and saved digitally via BrainWare software. The frequencies ranged from 2.5 kHz to 40 kHz, with an increment of 0.5 octave, and the levels ranged from 15 to 50 dB SPL, with an increment of 5 dB plus 50 to 90 dB SPL with an increment of 10 dB. Stimuli were presented randomly with a 50 ms interstimulus interval. The ABR receptive fields were recorded twice in each mouse, i.e., before and immediately after the TE_65_ (*n* = 20). To observe the time course of recovery after the TE_65_, additional mice (*n* = 10) were tested at pre-exposure and 1, 3, 4, 5, and 6 h post-exposure.

### 2.5. Data Processing

The ABR data were read out and displayed using SoundCode, a custom data processing software that allowed us to measure an individual ABR waveform and characterize the ABR receptive field, including best frequency and minimum threshold. Recording traces contaminated by artifacts were all excluded from the trace averaging for delineating the ABR wave. Wave peak latencies were used to identify different waves. Four waves were typically identified in our recordings. Wave III presented as a merged wave III/IV. Waves I and II were prominent and were used to determine the best frequency and response threshold based on the ABR receptive field delineated by the series of ABRs in response to the tone set (with various frequencies and levels) as described above.

The minimum threshold of the ABR was defined as the lowest response threshold across all frequencies. The best frequency of the ABR was defined as the frequency at which the minimum threshold was achieved. Wave latency was the range from tone onset to wave peak time. Wave amplitude was the peak voltage subtracted from the voltage from the subsequent valley. Individual ABR waves were identified according to wave peak latencies in responses to the tone of the best frequency and 60 dB SPL; specifically, they were 1.5–2.4 ms for Wave I, 2.5–3.8 ms for Wave II, 3.9–5.5 ms for Wave III/IV, and 5.5–7.5 ms for Wave V [22].

### 2.6. Statistical Analysis

Data are presented as mean ± standard deviation (SD). Paired *t*-tests (two-tailed) were applied to compare the thresholds and wave amplitudes between pre- and post-TE_65_. Pearson’s correlation coefficient was calculated to assess the linear relationship of the changes between wave amplitude and latency. The significance of correlation coefficients was evaluated using a two-tailed *t*-test. A *p*-value < 0.05 was considered statistically significant.

## 3. Results

**Figure 1:**
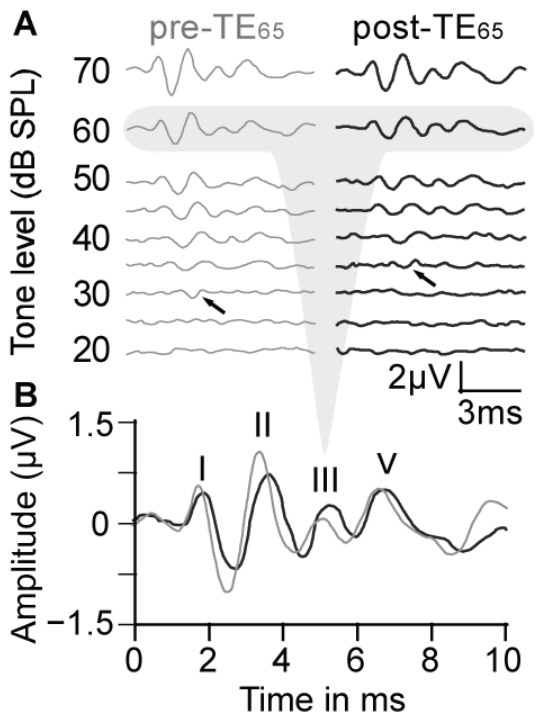
A typical example of an ABR waveform in response to tones with various frequencies and levels (**A**) and ABR waves in response to a tone at 60 dB SPL (**B**). Threshold increases following TE_65_ are indicated by black arrows in pre-TE_65_ (grey) and post-TE_65_ (black). Waves I, II, III, and V were identifiable in this ABR waveform.

Tone-induced ABR waveforms pre- and post-TE_65_ were similar in all mice and typically showed four distinct waves within a time window of 10 ms when the tone level was at 60 dB SPL or 70 dB SPL. As the tone level decreased, the number of visible waves also declined. Wave I/II exhibited the largest amplitudes and remained identifiable at the threshold levels. Wave amplitudes decreased and latencies increased as the tone level decreased (Figure 1). The best frequency of the ABR was either 10 kHz (*n* = 17) or 14.142 kHz (*n* = 13). The minimum threshold was 22.67 ± 5.37 dB SPL (*n* = 30) and fell within a range of 15 to 30 dB SPL.

### 3.1. ABR Threshold Increase and Its Frequency-Specificity

**Figure 2:**
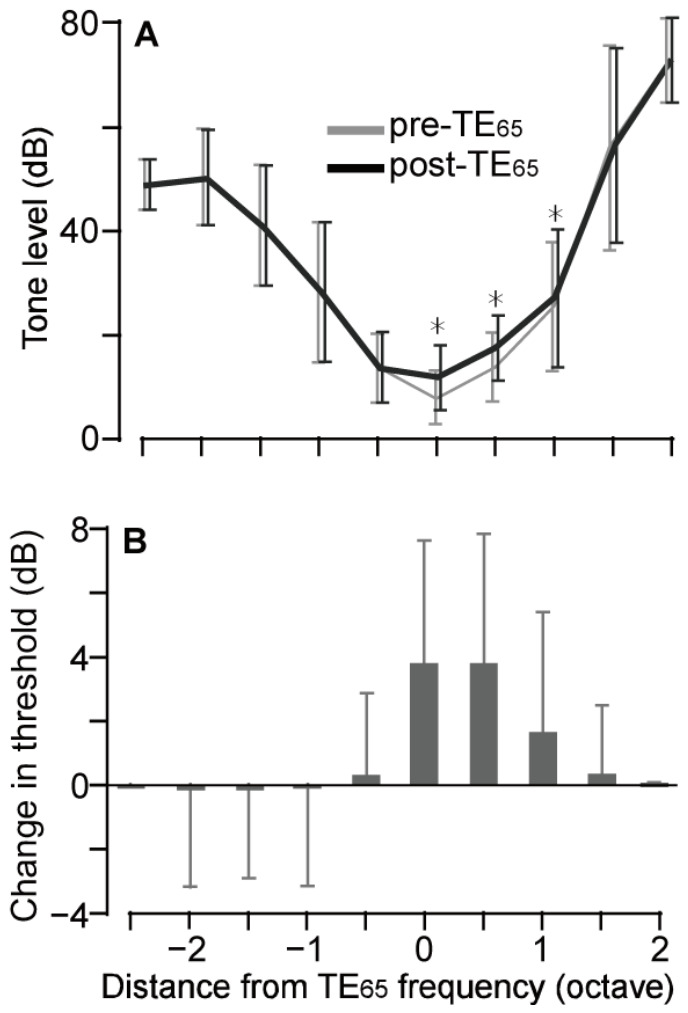
Frequency-dependent changes in ABR thresholds. (**A**). Comparisons of averaged ABR thresholds in response to the tones of different frequencies before and after TE_65_ (*n* = 30). Because the data are aligned to the TE_65_ frequency during averaging, that frequency is shown as 0 octave, and all other frequencies are displayed as their octave distance from TE_65_. (**B**). The differences in the thresholds (*n* = 30) across all frequencies before and after TE_65_. The thresholds in a range from TE_65_ frequency to 1 octave above are remarkably increased by TE_65_. * indicates the *p* value <0.05 (paired *t*-test).

TE_65_ increased the threshold in response to the exposure frequency in 18 out of 30 mice, with the largest threshold increase being 15 dB. On average, the threshold increased by 3.83 ± 3.80 dB (*p* < 0.001, *n* = 30), as shown in Figure 2B. When excluding the samples without threshold change, the averaged threshold elevation was 6.39 ± 2.92 dB, from 21.39 ± 5.09 dB SPL to 27.78 ± 6.24 dB SPL (*p* < 0.001, *n* = 18). Notably, in mice without threshold shifts, TE_65_ also significantly decreased the ABR wave amplitude at the level of the minimum threshold (0.28 ± 0.09 µV vs. 0.22 ± 0.10 µV, *p* = 0.008, *n* = 12). At the minimum threshold, one wave was typically identifiable, and this wave mostly represented Wave I in our recording. To illustrate the TE_65_-affected regions relative to the exposure frequency, ABR tuning curves from all mice were aligned to the exposure frequency, as shown in Figure 2A. TE_65_ appeared to increase the thresholds not only at the exposure frequency but also at higher frequencies within approximately one octave (Figure 2B). The greatest threshold increase was observed at 0.5 octaves above the exposure frequency (26.95 ± 6.67 dB SPL vs. 31.94 ± 6.22 dB SPL, *p* < 0.0001, *n* = 18). In contrast, TE_65_ had minimal effects on the thresholds at frequencies below the exposure frequency (Figure 2A,B).

### 3.2. Decrease in Amplitude and Increase in Latency of ABR Waves

**Figure 3:**
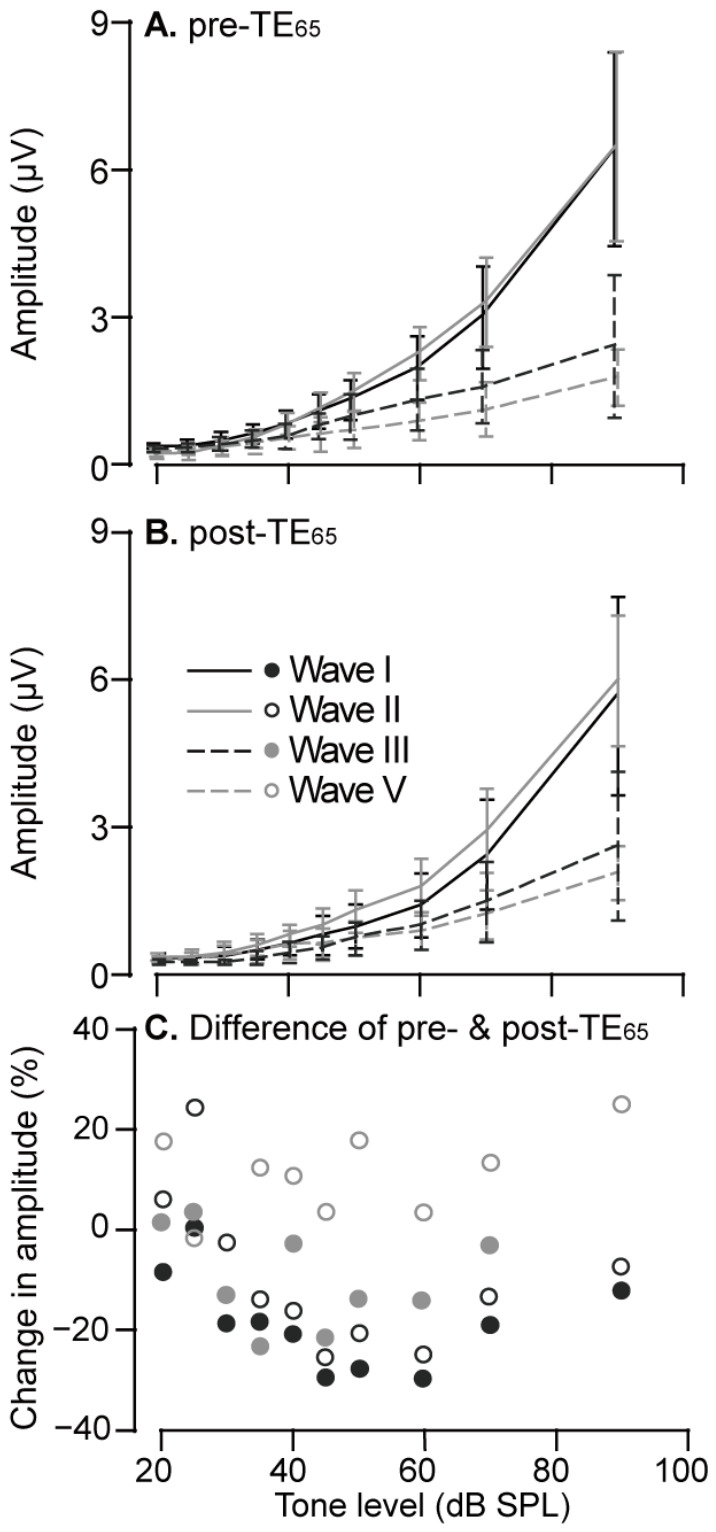
TE_65_-induced changes in the amplitudes across tone levels. The amplitudes of ABR waves before (**A**) and after (**B**) TE_65_ are plotted as the function of tone levels. They show that the patterns of amplitude-level functions were similar before and after TE_65_. The remarkable decrease in the amplitudes of Waves I–III occurred when the tone levels fell within a range of 45 to 60 dB SPL (**C**).

In general, the amplitudes of Waves I, II, III, and V changed as the function of tone level changed; the higher the tone level, the greater the impact on the amplitude. TE_65_ markedly altered ABR wave amplitudes, with Waves I/II consistently showing decreased amplitudes (Figure 1B). Across all 30 animals, including those with and without threshold shift, the level-amplitude function was remarkably steeper for Waves I and II than for Waves III and V (Figure 3A). TE_65_ did not alter the overall pattern of level-amplitude function (Figure 3B).

We further analyzed TE_65_-induced amplitude changes across tone levels. Changes in wave amplitudes were expressed as a percentage [100 × (pre-TE_65_ − post-TE_65_)/pre-TE_65_]. TE_65_ induced changes in the amplitudes of all four waves, forming a “U-shaped” pattern across 20–90 dB SPL (Figure 3C). Changes in Waves I–II were predominantly negative, indicating amplitude reductions across all tone levels. The greatest decrease occurred between 45 and 60 dB SPL, which represents more than a 20% decrease, with a maximum reduction of 28.5% at 60 dB SPL. For Wave III, amplitude decreases greater than 20% were observed at 35 dB SPL and 45 dB SPL, whereas amplitude increases were observed at the levels of 20, 25, and 90 dB SPL. In contrast, TE_65_-induced changes in Wave V amplitude were predominantly positive. This was notable given the general decrease in amplitudes of Waves I–III.

**Figure 4:**
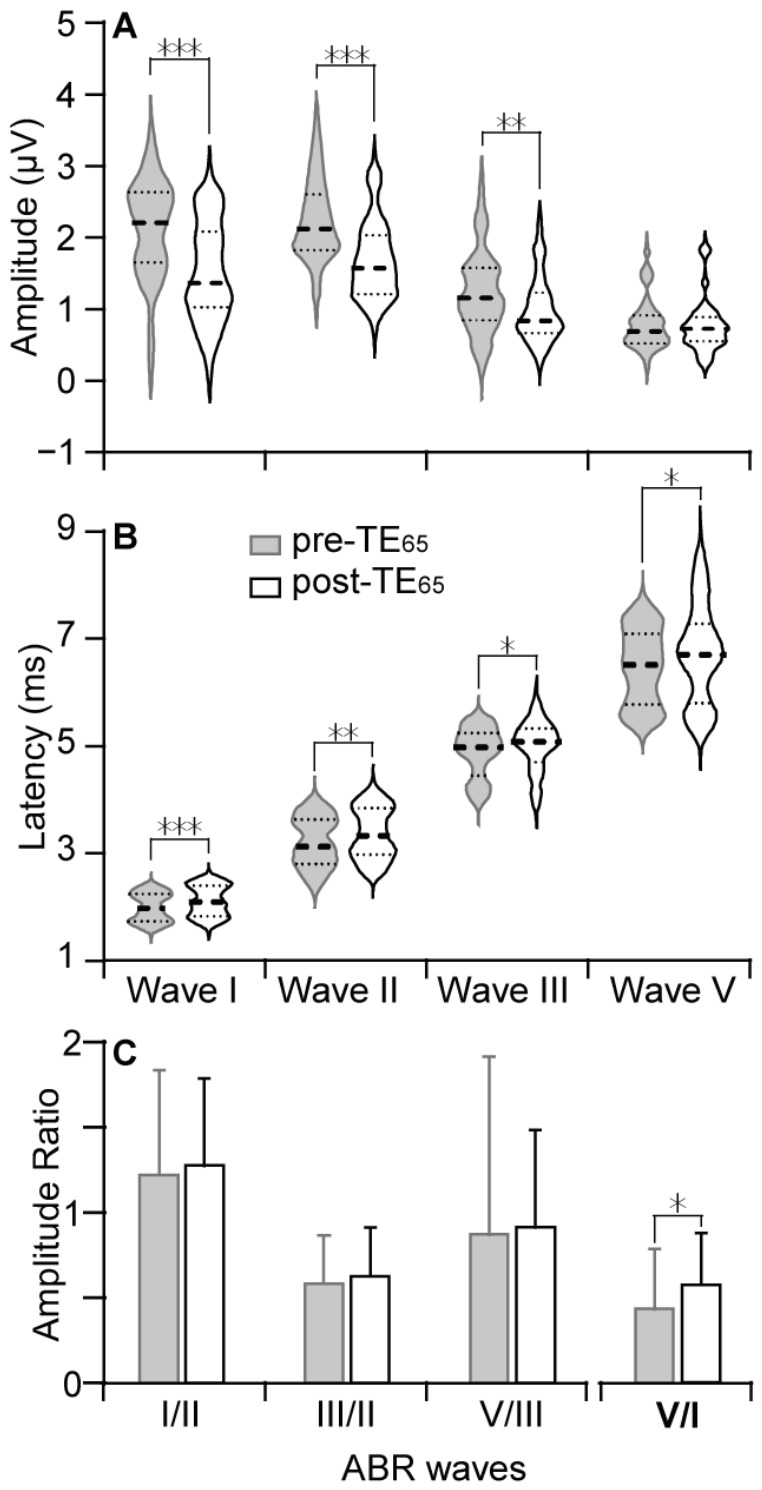
Comparison of the TE_65_-induced changes in the amplitudes and latencies of Waves I–V for the tone burst at the exposure frequency and 60 dB SPL. TE_65_ significantly decreased the amplitudes (**A**) and increased the latencies (**B**) of Waves I–III. A trend of amplitude increases from Wave I to Wave V was observed, as well as a significant increase in the ratio of Wave V to Wave I (**C**). In violin plots, the thick dash line represents the median, and light dash lines represent quartile. * indicates *p* <0.05, ** indicates *p* < 0.01, *** indicates *p* < 0.001.

Because the greatest decreases in the amplitude of Waves I and II occurred at 60 dB SPL, we compared the latency and amplitude of different waves at this tone level before and after TE_65_. As shown in Figure 4A, TE_65_ significantly reduced amplitudes in Wave I (2.14 ± 0.69 µV to 1.52 ± 0.67 µV, *p* < 0.001), Wave II (2.24 ± 0.54 µV to 1.68 ± 0.54 µV, *p* < 0.001), and Wave III (1.23 ± 0.57 µV to 0.98 ± 0.45 µV, *p* < 0.001). Wave V amplitude was not significantly affected by TE_65_. In contrast, TE_65_ significantly increased the latencies across all waves (Figure 4B): Wave I by 0.09 ± 0.27 ms (*p* < 0.001), Wave II by 0.16 ± 0.47 ms (*p* < 0.001), Wave III by 0.14 ± 0.47 ms (*p* < 0.05), and Wave V by 0.26 ± 0.80 ms (*p* < 0.05).

TE_65_-induced inter-wave effects were examined by amplitude ratios between waves. The ratios revealed a trend of consistent increase from Wave I to Wave V, and the ratio of Wave II to Wave I showed the largest increase. However, these increases were statistically insignificant (Figure 4C). We therefore compared the amplitude ratios of Wave V to Wave I; this ratio represents a summation of the amplitude differences among all waves. As expected, TE_65_ significantly increased the ratio of Wave V to Wave I (0.43 ± 0.35 vs. 0.57 ± 0.30, *p* < 0.01). The largest increase in the ratio of Wave II to Wave I may have been due to the significant decrease in the absolute amplitude of Wave I, while the increases among the other waves might signify the compensatory enhancement of the activities in the neural structures following the response of the auditory nerve.

### 3.3. Degraded Correlations of Amplitude and Latency Changes from Wave I to Wave V

**Figure 5:**
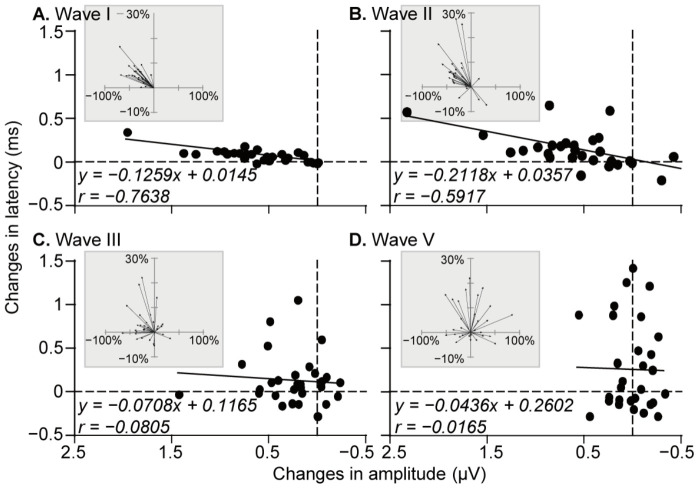
Correlations of TE_65_-induced decreases in the amplitude and increase in the latency of ABR waves. High correlations were observed in Waves I (**A**) and II (**B**) and poor correlations were observed in Waves III (**C**) and V (**D**). Trend lines (solid line) represent linear relationship between changes in amplitude and changes in latency. Insets: vectors show the percentage changes in the amplitude and latency.

Although the response latency is generally negatively associated with the response amplitude, we further examined the correlation of TE_65_-induced changes in the amplitude and latency of all waves. In Wave I, decreases in amplitude were consistently accompanied by increases in latency, showing a strong negative linear correlation (Figure 5A, *p* < 0.001). This relationship persisted in Wave II, with only two exceptions (Figure 5B, *p* < 0.001). More variability was observed in Waves III and V, where amplitude and latency changes did not consistently follow this pattern (Figure 5C,D). Consequently, correlations in these waves were no longer significant (*p* > 0.05). Vector plots (insets in Figure 5) illustrate the progressive degradation of this relationship from Wave I to Wave V.

### 3.4. Long-Lasting Effect of TE_65_

**Figure 6:**
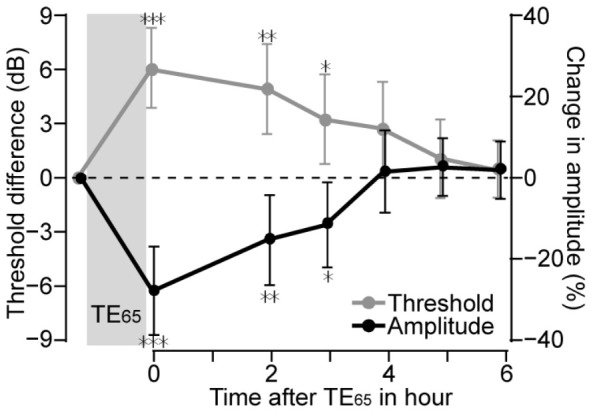
The time course of the changes in ABR threshold and Wave I amplitude after TE_65_. For both ABR threshold and Wave I amplitude, TE_65_-induced changes lasted at least 3 h after the exposure; these changes remained statistically significant. * indicates *p* <0.05, ** indicates *p* < 0.01, *** indicates *p* < 0.001.

The effects of the TE_65_ on the ABR were not transient but persisted for several hours. To assess the recovery, ABR thresholds and Wave I amplitudes were measured in 10 mice for up to 6 h post-exposure. As shown in Figure 6, maximal effects occurred immediately after TE_65_, including increases in ABR threshold and a reduction in Wave I amplitude at 60 dB SPL. On average, thresholds increased by 6.11 ± 2.20 dB (*p* = 0.0002, *n* = 10), and Wave I amplitude decreased by 27.20 ± 10.80% (*n* = 10, *p* < 0.0001). The changes were equivalent to those calculated on 30 animals, as presented above. The extent of TE_65_-induced changes gradually declined over time. Thresholds dropped at a rate of approximately 1.019 dB/hr and were close to pre-TE_65_ levels (19.44 ± 5.27 dB SPL vs. 16.67 ± 3.54 dB SPL, *p* = 0.054) at 6 h after tone exposure. Since the threshold levels were significantly higher than pre-TE_65_ levels for 3 h after tone exposure, we can conclude that the threshold increases persisted for this length of time. TE_65_-induced reductions in Wave I amplitudes at 60 dB SPL underwent a similar course of 3 h after tone exposure, and the amplitude decreases were statistically significant during this period. At 4 h after tone exposure, Wave I amplitude returned to and even slightly exceeded the pre-TE_65_ amplitude. Overall, both threshold and amplitude changes shared a recovery time course of approximately 3 h, although threshold recovery appeared slightly delayed.

## 4. Discussion

Our data show that TE_65_ increased ABR thresholds by up to 15 dB, based on measurements of the greatest ABR waves (typically Waves I/II). Importantly, the threshold increase was specific to the frequency of the exposed tone. Along with the threshold increase, significant decreases in amplitude and increases in latency were observed in Waves I–III. TE_65_-induced changes in response to the tones were greatest in the 45–60 dB SPL range and persisted for at least 3 h. Notably, the decrease in amplitude was negatively correlated with the increase in latency in Wave I. This correlation gradually degraded from Wave I through to Wave V. The effects of the TE_65_ on the ABR threshold and wave amplitude lasted for at least 3 h.

### 4.1. Input-Specificity of TE_65_-Caused Increase in ABR Threshold

Several studies using moderate narrow-band noise demonstrate that the auditory responses of cortical and subcortical neurons are altered or impaired mostly within the frequency range of exposure noise [23,24]. To characterize frequency-specific effects, this study employed a pure tone as the acoustic exposure so that the exposure energy was concentrated at a single frequency. Our ABR data show that significant threshold elevation occurred from the exposure frequency to the frequencies 1 octave above it, whereas changes in thresholds to frequencies below the exposure frequency were minimal. This suggests that the impact of tone exposure is restricted to the exposure frequency and its neighbouring higher frequencies. As is well-known, sound-triggered vibrations of the basilar membrane travel from the base (high frequency) toward the apex (low frequency). The displacement gradually increases until a maximum is reached at the segment corresponding to the stimulus frequency and then rapidly dissipates afterwards [25,26]. This theory of a sound wave travelling along the basilar membrane in the cochlea may describe the frequency-specificity of TE_65_-caused increases in ABR thresholds. However, the peak threshold shift occurred approximately 0.5 octaves above the exposure frequency, a pattern that cannot be fully explained by basilar membrane displacement alone. The asymmetric frequency distribution of threshold shifts aligns with classic single-neuron findings showing that maximal temporary threshold shift occurs when exposure is approximately half an octave below a neuron’s characteristic frequency [27]. Rather than a measurement artifact, the phenomenon of “half-octave shift” likely arises from intrinsic cochlear processing, particularly the nonlinear properties of cochlear amplification [28]. Under such conditions, regions basal to the characteristic place may experience disproportionately greater excitation, thereby increasing their vulnerability to functional impairment. Due to the inherent limitations of ABR, further clarification will require direct measurements of inner ear function, such as compound action potential (CAP), following TE_65_.

### 4.2. Possible Mechanisms Underlying ABR Threshold Increase

Since ABR Wave I primarily reflects tone-evoked neural activity of auditory nerve terminals in the inner ear, the observed reduction in amplitude and prolongation of latency in Wave I suggest that TE_65_-induced ABR threshold increases originate mainly from impaired sensory transduction in the cochlea. This interpretation is supported by evidence showing that exposure of rats to a narrow-band noise at 65 dB SPL significantly decreased CAP amplitude and increased CAP thresholds [21].

Although no direct evidence of cochlear alterations was obtained in the present study, several established cochlear mechanisms may help explain the TE_65_-induced changes observed in ABR measures. It is well established that hearing loss resulting from high and moderate noise exposure can result in dysfunction of outer hair cells, inner hair cells, or both [29]. Outer hair cells, serving as a biological amplifier, play a crucial role in hearing sensitivity [30,31], and their function can be assessed indirectly via otoacoustic emissions. Inner hair cells, serving as a transducer, convert mechanical energy from basilar membrane vibrations into electrical signals that trigger action potentials in the auditory nerve via ribbon synapses [32,33]. It is generally believed that exposure to loud noise can destroy hair cells and that even moderate noise (e.g., 100 dB SPL) leads to the impairment of hair cells, including the loss of ribbon synapses [34,35]. These impairments are directly associated with hearing deficits [8,36]. Gannouni and colleagues provided a detailed understanding of inner ear damage in rats that were exposed to narrow-band noises of either 70- or 85-dB SPL (6 h/day for 3 months) [37]. Under transmission electron microscopy, obvious deformation/destruction of the organ of Corti is evidenced. These noise-induced damages include, but are not limited to, stereocilia fusion/disorganization, cell body perforation, loss of cell organelles, and cytoplasmic vacuoles in both outer and inner hair cells in limited basilar membrane segments, which are likely due to frequencies carried in the exposed noise. The number of spiral ganglion cells is also decreased following noise exposure. It is worth noting that these impairments are more serious in rats exposed to 85 dB SPL as opposed to 70 dB SPL.

Another possible mechanism underlying the observed reduction in amplitude is the enhancement of the medial olivocochlear reflex. Previous studies have shown that noise exposure strongly activates the MOC efferent system and can lead to sustained or strengthened suppression of cochlear gain, evidenced by the reduction in the distortion product otoacoustic emission [38,39]. It is therefore plausible that the reduced response amplitude observed in the present study reflects an upregulated medial olivocochlear-mediated inhibitory influence on outer hair cell function, which, in turn, diminishes basilar membrane amplification and auditory nerve output. Such efferent-driven modulation may persist beyond the immediate exposure period, contributing to the altered cochlear responses even in the absence of overt hair cell damage.

Taken together, these findings suggest that the changes observed in the present study may be attributed to a combination of peripheral dysfunction and efferent modulation, with both factors likely contributing to the altered cochlear responses. One limitation of the present study is that the acoustic stimulus used was a continuous pure tone, which represents an artificial exposure condition and does not fully reflect the complexity of real-world acoustic environments. Occupational and environmental noise exposures are often characterized by non-Gaussian temporal structures and fluctuating acoustic properties that may produce different auditory effects compared with continuous tonal stimulation [40]. Therefore, further experiments using more ecologically relevant acoustic exposures are required to better understand the auditory consequences of moderate-level sound exposure under real-world conditions.

### 4.3. Functional Alteration of the Low Brainstem

Due to impairments in the inner ear, altered inputs of the auditory nerve to the brain can lead to functional changes in the central auditory system. Previous studies have documented that moderate noise (around 100 dB SPL) exposure largely changes the neural presentation and processing of auditory information in the auditory cortex [41,42,43,44,45]. Although related knowledge in the inferior colliculus remains limited, it appears that prolonged exposure to moderate noise causes changes in auditory information processing from the cochlear nucleus to the inferior colliculus [46,47,48,49]. In contrast, exposure to low-level noise (65 dB SPL for 4 weeks) does not appear to produce detectable changes in the inferior colliculus, despite reductions in cochlear CAP amplitude [21].

ABR studies allow for the assessment of electrical events from the auditory nerve (Wave I) to the inferior colliculus (Wave V) simultaneously in one recording. Our data show a significant decrease in the amplitude of Waves I–III but not in the amplitude of Wave V. This is supported by previous investigations [47,50]. A novel finding in this study, by analyzing the change in the amplitude and latency of different waves, is the progressive degradation of the correlation between amplitude and latency changes from Wave I through to Wave V (Figure 5). This degraded correlation suggests that quick neural compensation or adaptation may occur in the low brainstem in response to TE_65_-induced peripheral deficits. Considering that ABR sampling was performed immediately after 1 h of tone exposure in this study, it would be interesting to investigate in what form and how the degraded correlation between amplitude and latency is presented when tone exposure is repeated or extended for days, weeks, and months.

### 4.4. Clinical Implications for HHL Diagnosis

Normal hearing has a range from 0 dB HL to 20 dB HL, and, according to WHO standards, hearing loss is defined as a diagnostic hearing threshold of higher than 20 dBA [51]. HHL is diagnosed because an individual presents with hearing difficulties but has a normal hearing threshold on routine audiograms. One possible explanation is that individuals with HHL have elevated hearing thresholds that still fall within the normal range based on current clinical guidelines.

Unlike the over 20 dB threshold shifts typically observed following loud noise exposure [52,53,54,55], moderate-noise-induced threshold increases are relatively subtle, e.g., ~6 dB in this study or ~8 dB in Liu’s study [21]. Therefore, it is not surprising that audiologists inform individuals reporting mild hearing difficulties (e.g., a few dB increase) that their hearing remains within the ‘normal’ range. Although additional research is needed before clinical implementation, our findings suggest that regular ABR testing (e.g., annually) in individuals with prolonged exposure to even moderately noisy environments may help detect early declines in hearing sensitivity. The degraded correlation of amplitude vs. latency from Wave I to Wave V may represent a potential biomarker for early detection.

## 5. Conclusions

Our study demonstrates that exposure to a pure tone for 1 h that corresponds to the low levels of a normal conversation may lead to significant changes in ABR in a frequency-specific manner. These ABR findings help to confirm some degree of impairment in the inner ear. From this, we can infer that ‘safe’ sound may not be necessarily safe but can potentially increase hearing threshold and alter auditory information processing in the brain. Furthermore, our findings contribute preliminary evidence that may inform future research on new approaches for audiologists to assess individuals who experience hearing difficulties, despite having hearing thresholds within the normal range.

## Data Availability

The raw data supporting the conclusions of this article will be made available by the authors upon request.
